# Transarterial chemoembolization with miriplatin vs. epirubicin for unresectable hepatocellular carcinoma: a phase III randomized trial

**DOI:** 10.1007/s00535-017-1374-6

**Published:** 2017-08-01

**Authors:** Masafumi Ikeda, Masatoshi Kudo, Hiroshi Aikata, Hiroaki Nagamatsu, Hiroshi Ishii, Osamu Yokosuka, Takuji Torimura, Manabu Morimoto, Kenji Ikeda, Hiromitsu Kumada, Tosiya Sato, Ikuko Kawai, Toru Yamashita, Hiroshi Horio, Takuji Okusaka, Miriplatin TACE Study Group

**Affiliations:** 1 Department of Hepatobiliary and Pancreatic Oncology, National Cancer Center Hospital East, 6-5-1 Kashiwanoha, Kashiwa, Chiba, 277-8577 Japan; 2 Department of Gastroenterology and Hepatology, Faculty of Medicine, Kindai University, Osaka, Japan; 3 Department of Gastroenterology and Metabolism, Hiroshima University, Hiroshima, Japan; 4 Department of Hepatology, Yame General Hospital, Fukuoka, Japan; 5 Hepatobiliary and Pancreatic Section, Gastroenterological Division, The Cancer Institute Hospital of JFCR, Tokyo, Japan; 6 Department of Gastroenterology and Nephrology, Chiba University, Chiba, Japan; 7 Division of Gastroenterology, Department of Medicine, Kurume University School of Medicine, Fukuoka, Japan; 8 Gastroenterological Center, Yokohama City University Hospital Medical Center, Kanagawa, Japan; 9 Department of Hepatology, Toranomon Hospital, Tokyo, Japan; 10 Department of Biostatistics, Kyoto University School of Public Health, Kyoto, Japan; 11 Sumitomo Dainippon Pharma Co., Ltd, Osaka, Japan; 12 Department of Hepatobiliary and Pancreatic Oncology, National Cancer Center Hospital, Tokyo, Japan

**Keywords:** Carcinoma, Hepatocellular, Chemoembolization, Therapeutic, Miriplatin, Epirubicin, Randomized controlled trial

## Abstract

**Background:**

This prospective study investigated the superiority of transarterial chemoembolization (TACE) with miriplatin over TACE with epirubicin regarding overall survival (OS) in patients with unresectable hepatocellular carcinoma (HCC).

**Methods:**

Patients with unresectable HCC were randomized 1:1 to receive TACE with miriplatin or epirubicin in lipiodol. The primary endpoint was OS; secondary endpoints were percentages of patients who achieved treatment effect (TE) 4 (100% necrotizing effect or tumor reduction), duration of time to TACE failure, and adverse events (AEs). OS was compared using a stratified log-rank test adjusted for clinical stage, Child–Pugh class, and institution.

**Results:**

Of 257 patients enrolled from August 2008 to August 2010, 247 were analyzed for efficacy and toxicity (miriplatin, *n* = 124; epirubicin, *n* = 123). Baseline characteristics were well balanced between the two groups. Median OS times were 1111 days for miriplatin and 1127 days for epirubicin (adjusted hazard ratio 1.01, 95% confidence interval 0.73–1.40, *P* = 0.946). TE4 rates were 44.4% for miriplatin and 37.4% for epirubicin. Median times to TACE failure were 365.5 days for miriplatin and 414.0 days for epirubicin. AEs of grade 3 or higher, including elevated aspartate aminotransferase (miriplatin, 39.5%; epirubicin, 57.7%) and elevated alanine aminotransferase (miriplatin, 31.5%; epirubicin, 53.7%), were less frequent in the miriplatin than the epirubicin group.

**Conclusions:**

OS after TACE with miriplatin was not superior to that after TACE with epirubicin; however, hepatic AEs were less frequent with miriplatin.

Clinical Trial Registration: JapicCTI-080632.

**Electronic supplementary material:**

The online version of this article (doi:10.1007/s00535-017-1374-6) contains supplementary material, which is available to authorized users.

## Introduction

The strategy for treatment of hepatocellular carcinoma (HCC) is determined by tumor characteristics and liver function, and may include resection, local ablative therapy, transarterial chemoembolization (TACE), chemotherapy, or radiotherapy. TACE is currently the mainstay of unresectable HCC and has been shown to significantly prolong survival in several randomized controlled trials compared with chemotherapy alone [1] or conservative treatment [2, 3]. Meta-analyses have also demonstrated a clear survival benefit of TACE for unresectable HCC [4, 5]. Therefore, TACE has been acknowledged as a palliative treatment for unresectable HCC. Conventional TACE, administered with lipiodol and chemotherapeutic agents followed by an embolic material such as a gelatin sponge particles, is widely used as standard treatment in Asian countries including Japan; TACE with drug-eluting beads is often used in Western countries. Epirubicin, doxorubicin, mitomycin C, and cisplatin are common in conventional TACE, but the effects on overall survival (OS) and complete response rate of these agents in this context are unknown. Epirubicin is currently approved for TACE in Japan, where it is most widely used with lipiodol to treat unresectable HCC [6].

Miriplatin, (SP-4-2)-[(1*R*,2*R*)-cyclohexane-1,2-diamine-*N*,*N*′]bis(tetradecanoato-*O*)platinum monohydrate, is a third-generation lipophilic platinum derivative developed to treat HCC via hepatic artery administration as a sustained-release suspension with lipiodol [7]. Miriplatin is retained in local tumors with lipiodol and slowly releases an active platinum drug for a persistent antitumor effect; little transfer occurs to the systemic circulation, and systemic adverse events (AEs) are reduced. A phase I study of miriplatin with lipiodol indicated a recommended dose of 20 mg/mL with 6 mL of lipiodol [8], and an early phase II study of miriplatin with lipiodol showed a promising anticancer effect with a mild toxicity profile in patients with unresectable HCC [9]. In a randomized late phase II study, the efficacy of miriplatin with lipiodol was similar to that of zinostatin stimalamer (SMANCS^®^) [10], another lipophilic anticancer agent used to treat unresectable HCC in Japan [11, 12]. Subsequently, miriplatin was approved as a chemolipiodolization agent in Japan in October 2009. A pilot study of TACE with miriplatin showed no severe AEs and a good antitumor effect in patients with HCC [13]. TACE with miriplatin is anticipated to be more effective and less toxic than conventional TACE with epirubicin. This study aimed to determine the superiority of TACE with miriplatin over TACE with epirubicin, in terms of OS, in patients with unresectable HCC.

## Methods

### Study design

This prospective, multicenter, open-label, randomized phase III trial was conducted between August 2008 and August 2010, and compared TACE with miriplatin vs. TACE with epirubicin in patients with unresectable HCC. The primary endpoint was OS. Secondary endpoints were the proportion of patients showing treatment effect (TE) 4 (100% necrosis or reduction of the treated tumor), time to TACE failure, and AEs. This study was registered with the Japanese Pharmaceutical Information Center (JapicCTI-080632) and was conducted in full accordance with the guidelines for Good Clinical Practice and the Declaration of Helsinki. Written informed consent was obtained from each participant; the protocol and any modifications were from an institutional review board for each participating site.

### Eligibility criteria

Included in the study were patients aged at least 20 years having histologically or clinically (e.g., angiography and computed tomography [CT]) diagnosed HCC; measurable disease (i.e., a lesion having at least 10 mm as its longest diameter, measurable in two dimensions with dynamic CT); tumor stains on dynamic CT (arterial phase); no indications for hepatectomy, percutaneous ethanol injection, percutaneous microwave coagulation, or radiofrequency ablation; tumor, lymph node, metastases (TNM) stage II or III by the classification of Liver Cancer Study Group of Japan (LCSGJ) (e.g., tumor size greater than 2 cm, multiple tumors, or both) [14, 15]; Child–Pugh class A or B; liver damage grade A or B (classified by ascites, serum bilirubin, albumin, indocyanine green retention at 15 min, and prothrombin time) [14, 15]; sufficient organ function; a white blood cell count of at least 3000/µL; a platelet count of at least 5.0 × 10^4^/µL; serum total bilirubin of less than 3.0 mg/dL; and an Eastern Cooperative Oncology Group performance status (ECOG PS) of 0–2.

Exclusion criteria were hypersensitivity to iodine-containing drug/contrast medium, gelatin-containing injection product or food, or epirubicin or platinum; thyroid disease requiring any treatments or renal failure requiring dialysis; history of myocardial infarction or arrhythmia requiring treatment; active concomitant cancer; obvious tumor thrombosis in the bile duct, portal vein, or hepatic vein; history of previous TACE; systemic chemotherapy; and history of treatment within 4 weeks prior to giving informed consent for this study.

### Treatment method

Eligible patients were temporarily registered and allocated to the miriplatin and epirubicin groups at a ratio of 1:1 with open-label, dynamic allocation before undergoing angiography. The final registration was completed by each participating investigator after confirmation of the following conditions by angiographic findings: intrahepatic lesions showing tumor staining that were fed by an appropriate artery for catheter insertion; no evidence of tumor thrombosis in the main portal or hepatic vein; and no evidence of severe intrahepatic arterio-venous shunt. Stratification factors were TNM stage of LCSGJ, Child–Pugh class, and institution. TACE was performed using the Seldinger technique. The dose of anticancer agents was determined according to tumor size. Maximum doses were defined as 120 mg/person for miriplatin (MIRIPLA^®^; Sumitomo Dainippon Pharma, Japan) and 60 mg/person for epirubicin (Farmorubicin^®^; Pfizer, USA). Patients allocated to the miriplatin group were given miriplatin suspended in 6 mL of lipiodol (20 mg/mL). Patients allocated to the epirubicin group were given epirubicin in 6 mL of solution suspended with 6 mL of lipiodol (10 mg/mL). Embolization was achieved using 1- or 2-mm porous gelatin particles (Gelpart^®^, Nippon Kayaku, Japan) from the feeding artery (both groups) according to the tumor size and vascular diameter (upper limit 80 mg/session). Tumor response was evaluated by dynamic CT at 5 and 12 weeks after each TACE session. TACE was repeated when the accumulation of lipiodol in the treated tumor was insufficient and tumor staining or new lesions were seen by follow-up dynamic CT evaluation. During periods of treatment, TACE was repeated on an as-needed basis until discontinuation criteria were met or a maximum of 3 years after the first session of TACE; TACE was administered repeatedly as indicated at minimum intervals of 4 weeks. The criteria for administration of subsequent treatments were as follows: Child–Pugh class A or B; liver damage grade A or B; sufficient organ function; ECOG PS of 0–2; no hypersensitivity to iodine-containing contrast medium, gelatin, epirubicin, or platinum; and no obvious tumor thrombosis in the bile duct or portal/hepatic veins. Discontinuation of treatment occurred when less than 50% of the necrotizing effect was achieved in the target lesion and enlargement of at least 25% occurred in the treated tumor; or when sufficient recovery from previous TACE to meet the criteria of subsequent TACE could not be expected. Completion of protocol treatments with TACE was defined as not meeting discontinuation criteria or a maximum of 3 years after the first session of TACE. After termination of protocol treatment, any other anticancer treatments, including hepatic arterial infusion chemotherapy, systemic chemotherapy, and, radiotherapy, could be administered.

### Efficacy and safety evaluation

OS time was calculated as the period from the first day of administration until death from any cause or last follow-up. The TE after the first administration was judged using the response criteria proposed by LCSGJ [15], in which lipiodol accumulation in the tumor is regarded as an indication of necrosis. TE was defined as follows: TE4, 100% necrosis or 100% reduction in size of all targeted tumors; TE3, at least 50% or less than 100% of tumor necrotizing effect or tumor size reduction rate, respectively; TE2, effects other than TE3 or TE1; TE1, greater than 25% tumor enlargement, regardless of the necrotizing effect. The tumor responses were evaluated in a blinded manner by an external committee for efficacy evaluation. Time to TACE failure was defined as the period from the first day of TACE administration to the completion or discontinuation of the treatment protocol. If the date of completion or discontinuation could not be confirmed, the date of the final hospital visit was used as the end date of completion or discontinuation. Specified laboratory tests were performed at 3 and 7 days, then 2, 3, 5, and 12 weeks, and AEs were evaluated throughout TACE treatment using the Common Terminology Criteria for Adverse Events (CTCAE) v3.0. OS and time to TACE failure was calculated using the Kaplan–Meier method.

### Statistical analysis

The primary analysis compared the OS in the miriplatin group with that of the epirubicin group with a stratified log-rank test adjusted for clinical stage, Child–Pugh class, and institution. A hazard ratio (HR) was generated with a stratified Cox regression model. A subgroup analysis of OS by patient background was also conducted. The study population was defined as the full analysis set (FAS), including any patients who received at least one course of the study treatment. The 2-year survival rate with miriplatin was estimated to be 76–80%, as informed by a 2-year survival rate of 75.9% in a randomized phase II study of miriplatin without embolization [10] and a somewhat expected increase in survival when used in combination with embolization. The 2-year survival rate of patients treated with epirubicin was assumed to be 63%, as informed by results of a study using doxorubicin [3] and a Japanese multicenter prospective cohort study [16]. Assuming a 2-year survival rate of 76–80% in patients treated with miriplatin and 63% in those treated with epirubicin, a total of 200 patients were needed to verify the superiority of miriplatin over epirubicin using a two-sided significance level of 5% and 80% power. To account for the potential loss of patients to follow-up, the number of planned patients enrolled was set at 220. Statistical Analysis System (SAS Institute Inc., Cary, NC, USA) version 9.2 was used for all statistical analyses.

## Results

### Patient disposition and characteristics

**Fig. 1:**
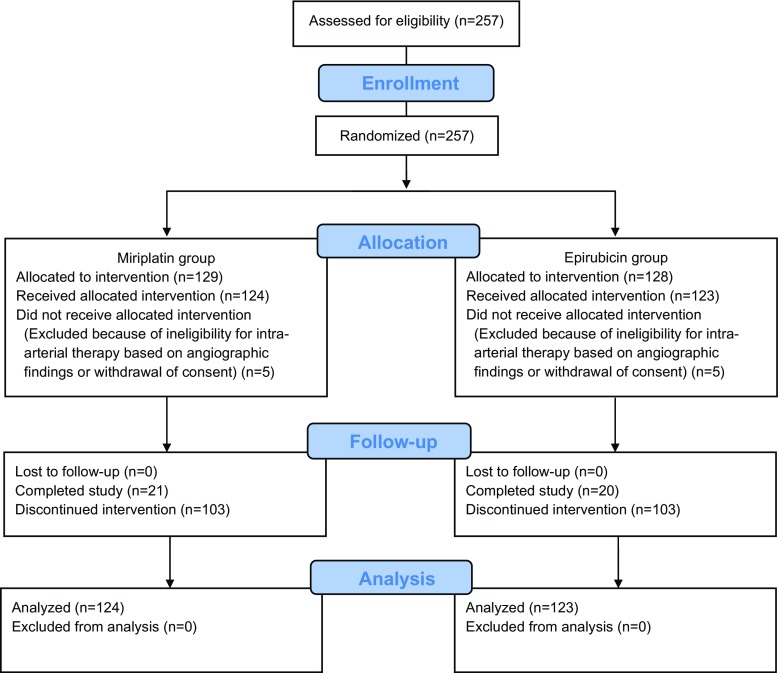
Patient allocation

Of the 257 patients enrolled at 29 participating hospitals, 129 and 128 patients were allocated to the miriplatin and epirubicin groups, respectively. Of these, 124 and 123 patients in the miriplatin and epirubicin groups, respectively, were included in the FAS (Fig. 1). Baseline characteristics were well balanced between the two groups (Table 1).

**Table 1:** Patient characteristics

	Miriplatin group (*n* = 124)		Epirubicin group (*n* = 123)	
	No.	%	No.	%
Sex				
Male	92	74.2	92	74.8
Female	32	25.8	31	25.2
Age, years				
Median	72		71	
Range	46–86		40–87	
ECOG performance status				
0	116	93.5	114	92.7
1	7	5.6	5	4.1
2	1	0.8	4	3.3
Hepatitis viral infection				
HBs antigen-positive	15	12.1	17	13.8
HCV antibody-positive	71	57.3	77	62.6
Child–Pugh classification				
A	104	83.9	105	85.4
B	20	16.1	18	14.6
Tumor stage				
I	1	0.8	0	0
II	57	46.0	56	45.5
III	66	53.2	67	54.5
Previous treatment before TACE				
Hepatic resection	23	18.5	25	20.3
Local ablation	24	19.5	39	31.5
Other	1	0.8	2	1.6
None	86	69.4	79	64.2
Maximum tumor size (mm)				
Median	30.5		27.0	
Range	10.0–127.0		10.0–137.2	
No. of tumors				
Single	24	19.4	24	19.5
Multiple	100	80.6	99	80.5
Tumor distribution				
Single-segment	42	33.9	46	37.4
Multi-segment	82	66.1	77	62.6
AFP (ng/dL)				
Median	25.1		22.8	
Range	1–71,180		2–82,739	

*AFP* alpha-fetoprotein, *HBs* hepatitis B surface, *HCV* hepatitis C virus, *ECOG* Eastern Cooperative Oncology Group

Mean numbers of TACE sessions following the protocol were 2.1 and 2.2 in the miriplatin group and epirubicin group, respectively. Median total doses of drugs administered were 120.0 mg and 61.6 mg in the miriplatin group and epirubicin group, respectively. The protocol was discontinued in 103 patients in each group. The need for other treatment for residual or recurrent HCC was the most frequent reason for discontinuation, which was applied to 66 miriplatin patients (53.2%) and 67 epirubicin patients (54.5%) in the epirubicin group.

After termination of protocol treatment, 95 patients in the miriplatin and 96 in the epirubicin group underwent the following treatments: hepatic resection (zero and one patient, respectively), percutaneous ethanol injection (one and one, respectively), TACE with miriplatin (17 and 14, respectively), TACE with epirubicin (38 and 34, respectively), and TACE with another drug (11 and 15, respectively).

### Efficacy analysis

**Fig. 2:**
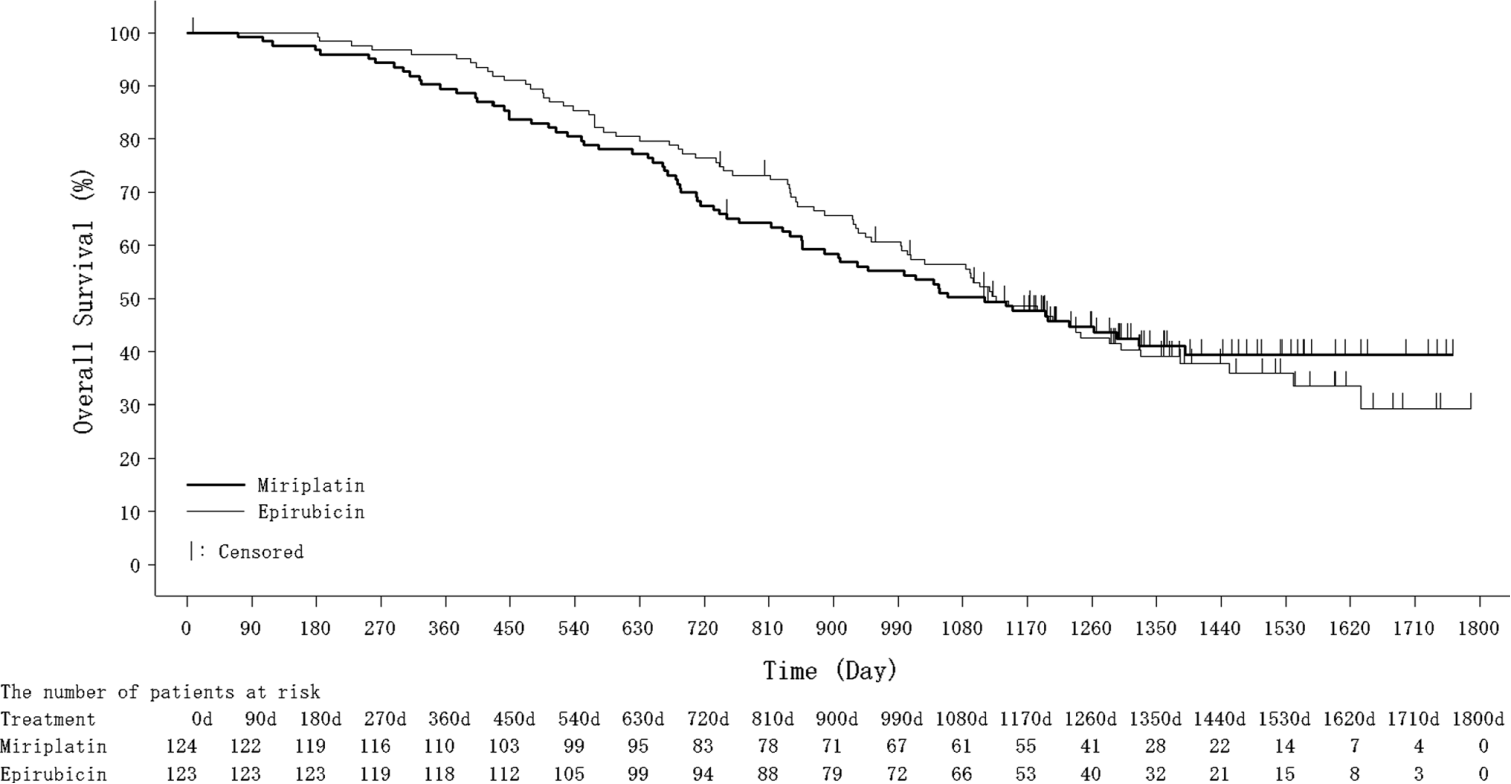
Overall survival rates in the miriplatin and epirubicin groups

**Fig. 3:**
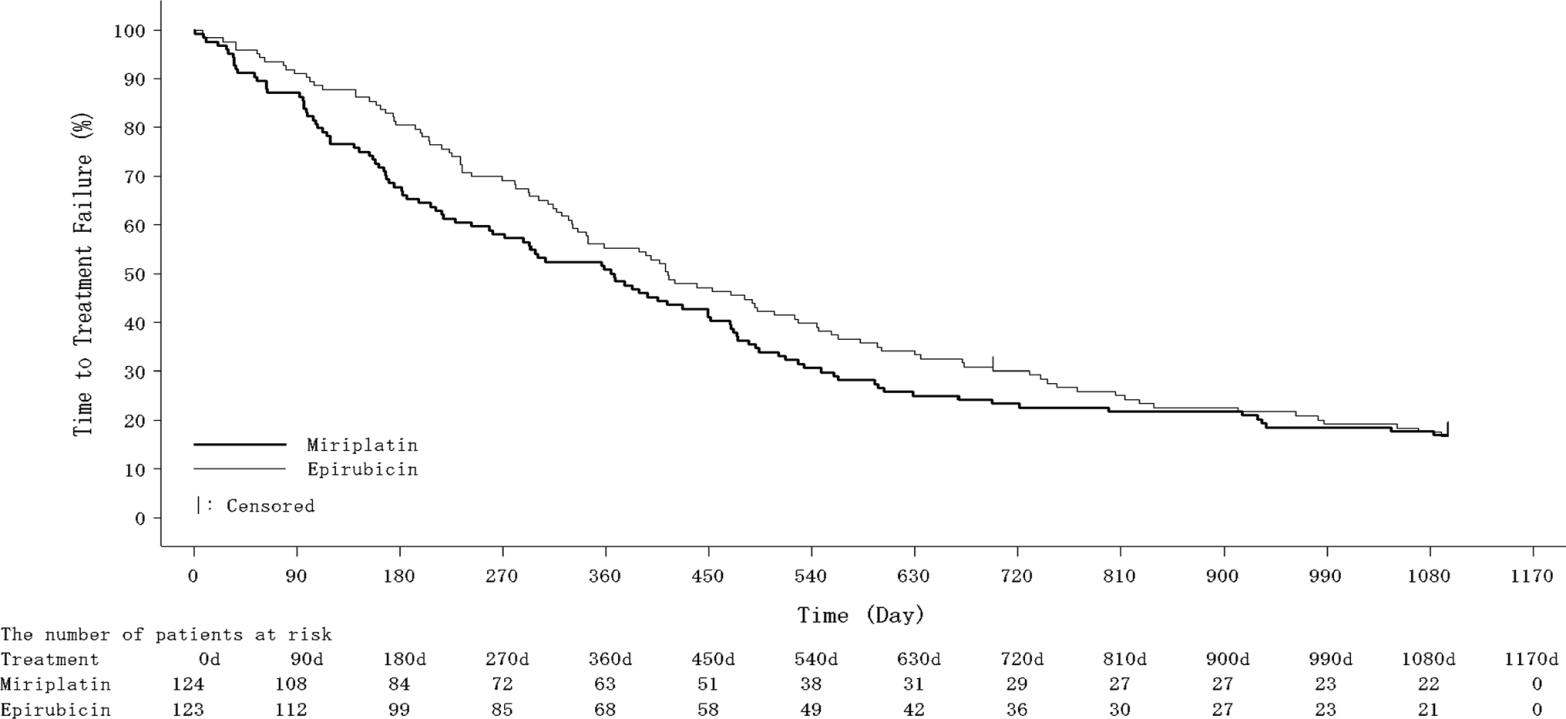
Time to TACE failure in the miriplatin and epirubicin groups

At the time of final analysis, 71 and 75 patients had died in the miriplatin and epirubicin groups, respectively. The median survival time and 2-year/3-year survival rates were 1111 days (miriplatin group; 95% confidence interval [CI] 888–1390) vs. 1127 days (epirubicin group; 95% CI 995–1300), 67% (miriplatin group; 95% CI 58–75) vs. 76% (epirubicin group; 95% CI 68–83), and 50% (miriplatin group; 95% CI 41–59) vs. 53% (epirubicin group; 95% CI 44–61), respectively (Fig. 2). The predefined stratified HR for OS by the Cox model adjusted for clinical stage and Child–Pugh class for miriplatin to epirubicin was 1.01 (95% CI 0.73–1.40), and the *P* value by log-rank test for the comparison of OS in the two groups gave a two-sided *P* value of 0.946. After the first session of TACE, TE4 was observed in 55 patients (44.4%) in the miriplatin group and 46 patients (37.4%) in the epirubicin group (*P* = 0.184). The median time to TACE failure was 365.5 days (95% CI 258–449) in the miriplatin group and 414.0 days (95% CI 335–507) in the epirubicin group (*P* = 0.250) (Fig. 3).

**Fig. 4:**
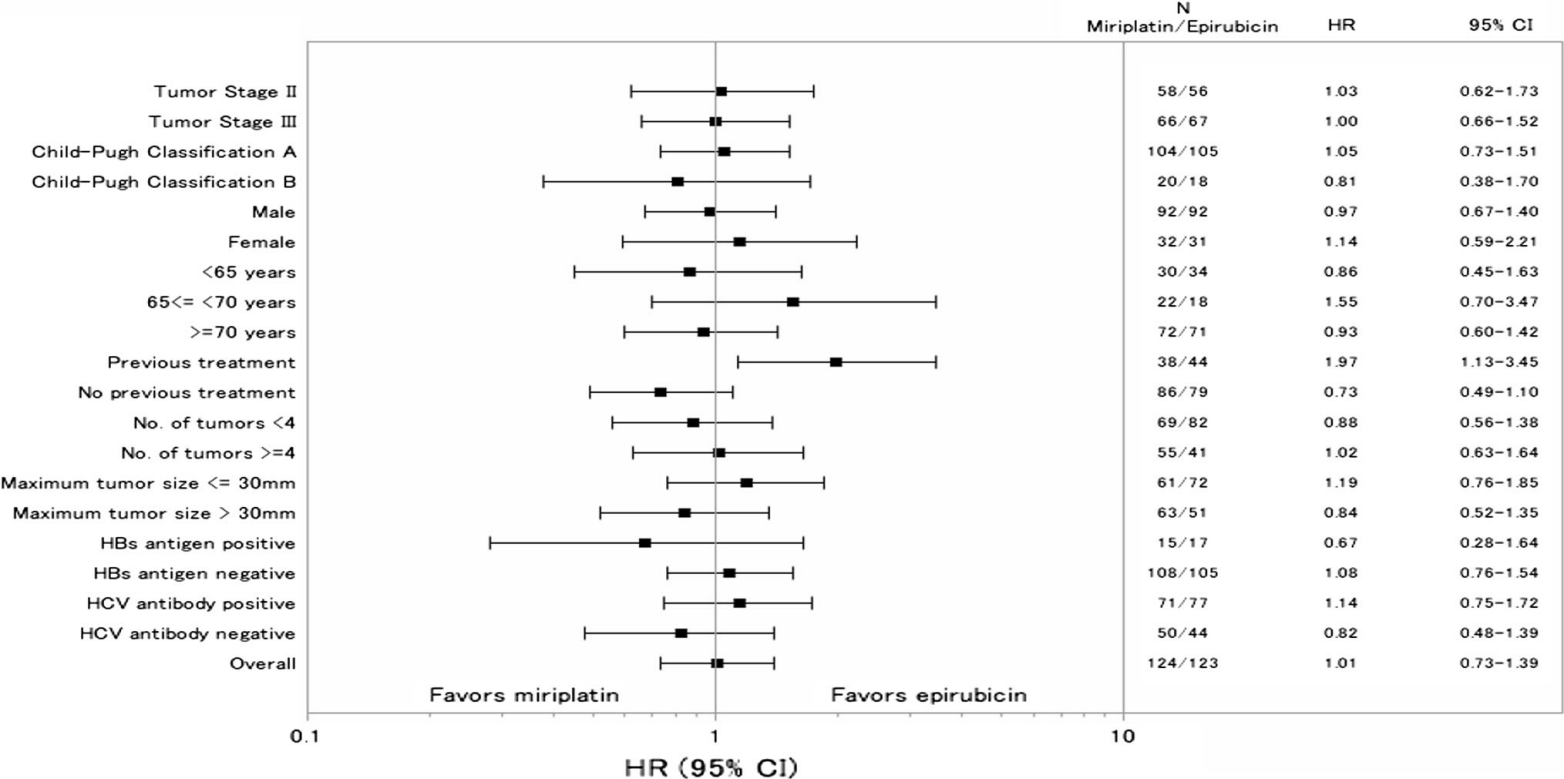
Subgroup analysis of overall survival rates in the miriplatin and epirubicin groups

In the prespecified subgroup analysis of OS by patient background, the OS in patients in the epirubicin group who had previous HCC treatment was longer than that of similarly treated patients in the miriplatin group (Fig. 4). No significant differences in OS between the groups were seen in the subgroup analysis.

### Adverse events

Table 2 shows the AEs in both groups for all protocol sessions of TACE. Symptoms of so-called post-embolization syndrome, such as fever, abdominal pain, and nausea, were frequently observed in both groups (Table 2). Decreased white blood cell count, fever, and increased alanine aminotransferase (ALT) were observed more frequently in the epirubicin group than the miriplatin group. Severe AEs of grade 3 or higher developed in 94 (75.8%) and 106 (86.2%) patients in the miriplatin and epirubicin groups, respectively; among these, at least grade 3 increased ALT and increased aspartate aminotransferase (AST) were observed less frequently in the miriplatin group (ALT, 31.5%; AST, 39.5%) than in the epirubicin group (ALT, 53.7%; AST, 57.7%) (AST, *P* = 0.005; ALT, *P* < 0.001). Clinically significant treatment-related serious AEs were cholangitis and liver abscess (one patient each) in the miriplatin group and hemorrhagic gastric ulcer, bacillemia, and septic shock (one patient each) in the epirubicin group. One treatment-related death due to an HCC rupture with hemorrhagic ascites on day 48 after the sixth TACE session occurred in the epirubicin group; no treatment-related death occurred in the miriplatin group. Almost all severe AEs developed initially following the first session of TACE; no cumulative AEs developed in this series. No hepatic injuries necessitating discontinuation occurred in the miriplatin group, whereas hepatic failure and bile duct stenosis each occurred in one patient in the epirubicin group.

**Table 2:** Adverse events for all protocol sessions of TACE according to treatment group

	Miriplatin group (*n* = 124)				Epirubicin group (*n* = 123)				*P* value*
	All grades		Grade ≥3		All grades		Grade ≥3		Grade ≥3
	*n*	%	*n*	%	*n*	%	*n*	%	
Hematological toxicity									
Eosinophil count increased	108	87.1	1	0.8	47	38.2	0	0	1.000
Platelet count decreased	76	61.3	14	11.3	85	69.1	20	16.3	0.274
Neutrophil count increased	56	45.2	0	0	58	47.2	0	0	–
White blood cell decreased	55	44.4	1	0.8	76	61.8	10	8.1	0.005
Neutrophil decreased	54	43.5	11	8.9	58	47.2	16	13.0	0.316
Hemoglobin decreased	70	56.5	1	0.8	68	55.3	3	2.4	0.370
White blood cell increased	52	41.9	0	0	45	36.6	0	0	–
Non-hematological toxicity									
Fever	117	94.4	2	1.6	123	100.0	1	0.8	1.000
Abdominal pain	80	64.5	1	0.8	94	76.4	3	2.4	0.370
Nausea	55	44.4	0	0	67	54.5	1	0.8	0.498
ALT increased	103	83.1	39	31.5	114	92.7	66	53.7	<0.001
AST increased	103	83.1	49	39.5	109	88.6	71	57.7	0.005
Glycemia increased	102	82.3	22	17.7	84	68.3	14	11.4	0.207
Hypoalbuminemia	97	78.2	1	0.8	98	79.7	0	0	1.000
Hyponatremia	77	62.1	6	4.8	63	51.2	9	7.3	0.439
Blood bilirubin increased	74	59.7	3	2.4	84	68.3	7	5.7	0.216

Grading according to the Common Terminology Criteria for Adverse Events, v3.0

*ALT* alanine aminotransferase, *AST* aspartate aminotransferase

* *P* values were calculated using two-sided Fisher’s exact test for grade ≥3 adverse events

Delayed fever of at least 1 week after treatment was a miriplatin-specific AE (Table S1). After the first session of TACE, the incidence of fever that developed within at most 7 days did not differ significantly between groups (miriplatin, 111 patients, 89.5%; epirubicin, 121 patients, 98.4%), while the incidence of fever that developed at least 8 days was significantly higher in the miriplatin group (80 patients, 64.5%) than in the epirubicin group (49 patients, 39.8%). However, this difference decreased as the number of TACE sessions increased. The incidence of eosinophilia following the first session of TACE was also significantly higher in the miriplatin group (105 patients, 84.7%) than the epirubicin group (28 patients, 22.8%). However, no clinical symptoms developed in patients with eosinophilia, and the incidence of eosinophilia also decreased as the number of TACE sessions increased.

## Discussion

Miriplatin is a structurally modified lipophilic platinum complex with improved affinity for lipiodol [7]. Miriplatin suspended in lipiodol showed favorable antitumor activities after hepatic arterial administration [17, 18] in animal models with hepatic tumors. Miriplatin is retained preferentially in liver tumors, which gradually release active platinum [8–10]; its low systemic distribution likely reduces systemic adverse effects. We conducted this study to elucidate the superiority of TACE with miriplatin over TACE with epirubicin as a combination chemotherapeutic regimen in patients with unresectable HCC.

Median survival was similar in the two groups, and superiority of TACE with miriplatin over TACE with epirubicin was not shown for the primary endpoint of OS. There was a crossover of treatments in this series: after termination of protocol treatment, 38 patients in the miriplatin group received TACE with epirubicin and 14 patients in the epirubicin group received TACE with miriplatin. Thus, we cannot exclude the possibility that these and other post-protocol treatments, such as hepatic resection and percutaneous ethanol injection, influenced OS. At the planning of this study, the survival rate of patients treated with miriplatin may have been overestimated and that of patients treated with epirubicin may have been underestimated. The expected 2-year survival rate of TACE with miriplatin in this phase III study was assumed to be 76–80%; however, the actual rate was only 67%. This discrepancy may be explained by differences in patient characteristics: more patients had multiple tumors or large tumor sizes in this study than in the randomized phase II study (Table S2). Conversely, the observed 2-year survival rate of 76% in the epirubicin group in this study was similar to that found in a prospective study of TACE in 99 unresectable HCC patients in Japan and Korea (75%) [19], and was more favorable than the estimate of 63%. Thus, the OS of patients recently treated with TACE plus epirubicin in Japan seems to be longer than that of patients receiving the same treatment in other countries or in earlier reported studies. In several randomized controlled studies comparing various chemotherapeutic agents combined with TACE for unresectable HCC, no survival benefit of the specific agent was demonstrated [20–22]. The combined use of chemotherapeutic agents may not influence TACE treatment.

In this study, the percentage of patients achieving TE4 following TACE did not differ significantly between the two groups (miriplatin, 44.4%; epirubicin, 37.4%); however, the CT evaluations may not have accurately reflected the extent of tumor necrosis because of the artifacts created by iodized oil. The complete response was reported to be 42% in the Asian TACE study mentioned above using anthracycline agents plus lipiodol with embolization [20]; a similar tumor response was observed in this study. Therefore, miriplatin and epirubicin were found to elicit equivalent antitumor effects after TACE, although an additional effect of embolization was observed compared with the TE4 rate (26.5%) following chemolipiodolization with miriplatin in a randomized phase II trial of miriplatin/lipiodol vs. zinostatin stimalamer/lipiodol [10]. Time to TACE failure tended to be shorter in the miriplatin than in the epirubicin group; however, this difference was not statistically significant. Because the stratified HR by the Cox model adjusted for clinical stage and Child–Pugh class was not calculated, the explanation for this remains unknown.

The tolerability of TACE with miriplatin in patients with liver dysfunction was favorable. Incidences of increased AST, ALT, and total bilirubin were lower in the miriplatin group than the epirubicin group. Most patients with unresectable HCC have liver cirrhosis, which is usually associated with compromised hepatic reserve. Therefore, the mild hepatotoxicity of TACE with miriplatin was beneficial for patients with unresectable HCC, considering that TACE was repeated. However, fever that developed at least 1 week after treatment and eosinophilia were also observed in the miriplatin group, mainly during the first sessions of TACE, and the incidence of these events decreased after more sessions of TACE with miriplatin. No findings were suggestive of anaphylactic reactions and no clinically serious events occurred, and the cause remains unknown. No other miriplatin-specific AEs occurred. The safety of TACE with miriplatin was consistent with the safety profile of miriplatin alone, and the combination was well tolerated.

This study has some limitations. First, miriplatin is a novel lipophilic platinum agent, and our results cannot be generalized to other platinum-based agents such as cisplatin. Second, miriplatin with TACE for treatment of HCC is currently approved only in Japan; therefore, these results cannot be generalized to populations in other countries. Finally, we did not use drug-eluting beads in this study, although these are often used in Western countries. The efficacy of miriplatin combined with drug-eluting beads has not been clarified in this study.

In conclusion, superiority of miriplatin over epirubicin for the OS endpoint was not demonstrated, although hepatic AEs were less frequent with miriplatin. It remains unclear which chemotherapeutic agent is most suitable for combined use with TACE for unresectable HCC.

## Electronic supplementary material

Below is the link to the electronic supplementary material.

Supplementary material 1 (PDF 48 kb)

Supplementary material 2 (PDF 56 kb)

Supplementary material 3 (PDF 12 kb)
