# Supplementary material for: Transarterial chemoembolization with miriplatin vs. epirubicin for unresectable hepatocellular carcinoma: a phase III randomized trial
Source: J Gastroenterol. 2017 Aug 1;53(2):281–90. doi: 10.1007/s00535-017-1374-6 (PMC5846877; doi:10.1007/s00535-017-1374-6)
Supplement: Supplementary file 1 — Supplementary material 1 (PDF 48 kb) [file 535_2017_1374_MOESM1_ESM.pdf]

Supplemental Table 1. Change in Fever Grade

	Miriplatin group (n = 124)				Epirubicin group (n = 123)			
	Grade 0		Grade ≥1		Grade 0		Grade ≥1	
	n	%	n	%	n	%	n	%
First administration in each group								
≤7 days	13	10.5	111	89.5	2	1.6	121	98.4
≥8 days	44	35.5	80	64.5	74	60.2	49	39.8
Second administration in each group								
≤7 days	8	11.0	65	89.0	3	4.1	71	95.9
≥8 days	57	78.1	16	21.9	54	73.0	20	27.0
Third administration in each group								
≤7 days	2	6.3	30	93.8	6	13.0	40	87.0
≥8 days	25	78.1	7	21.9	33	71.7	12	26.1
