# Supplementary material for: Transarterial chemoembolization with miriplatin vs. epirubicin for unresectable hepatocellular carcinoma: a phase III randomized trial
Source: J Gastroenterol. 2017 Aug 1;53(2):281–90. doi: 10.1007/s00535-017-1374-6 (PMC5846877; doi:10.1007/s00535-017-1374-6)
Supplement: Supplementary file 2 — Supplementary material 2 (PDF 56 kb) [file 535_2017_1374_MOESM2_ESM.pdf]

Supplemental Table 2. Patient Background in a Previous Phase II Trial and the Current Study (Miriplatin Group)

	Previous study* n = 83		Current study n = 124	
	No.	%	No.	%
Tumor number				
1	24	28.9	24	19.4
2	19	22.9	28	22.6
3	16	19.3	17	13.7
≥4	24	28.9	55	44.4
Maximum tumor size (mm)				
≤20	21	25.3	35	28.2
20 to <50	59	71.0	63	50.8
≥50	3	3.6	26	21.0

*Randomized phase II trial of miriplatin vs. zinostatin stimalamer.[1]

Reference

1. Okusaka T, Kasugai H, Ishii H, et al. A randomized phase II trial of intra-arterial chemotherapy using SM-11355 (Miriplatin) for hepatocellular carcinoma. Invest New Drugs. 2012;30:2015–2025.
