# Supplementary material for: Transarterial chemoembolization with miriplatin vs. epirubicin for unresectable hepatocellular carcinoma: a phase III randomized trial
Source: J Gastroenterol. 2017 Aug 1;53(2):281–90. doi: 10.1007/s00535-017-1374-6 (PMC5846877; doi:10.1007/s00535-017-1374-6)
Supplement: Supplementary file 3 — Supplementary material 3 (PDF 12 kb) [file 535_2017_1374_MOESM3_ESM.pdf]

Appendix : Investigators and study sites

Coordinating Investigators

Kenji Ikeda	Toranomon Hospital
Takuji Okusaka	National Cancer Center Hospital

Members of the Data and Safety Monitoring Board

Masaru Itakura	Surugadai Clinic, Medical Corporation Shun-ai-kai
Mariko Itsubo	The Jikei University School of Medicine
Shoji Fukushima	Faculty of Pharmaceutical Sciences, Kobe Gakuin University

Members of the Committee for Efficacy Evaluation

Hiroki Inoue	Foundation Jiaikai Imamura Hospital
Hirotoshi Nishida	Foundation Jiaikai Imamura Hospital

Investigators and Institutions

Masaharu Yoshikawa	Chiba University Hospital
Fumihiko Kanai	Chiba University Hospital
Osamu Yokosuka	Chiba University Hospital
Masafumi Ikeda	National Cancer Center Hospital East
Kohei Nakachi	National Cancer Center Hospital East
Hiroshi Ishii	Cancer Institute Hospital of JFCR
Keiko Tatemoto	Tokyo Women’s Medical University Hospital

Takuji Okusaka	National Cancer Center Hospital
Yoshihiro Kubokawa	Juntendo University
Masafumi Suyama	Juntendo University
Jinkan Sai	Juntendo University
Kenji Ikeda	Toranomon Hospital
Hiromitsu Kumada	Toranomon Hospital
Akihisa Miyazaki	Juntendo University Nerima Hospital
Manabu Morimoto	Yokohama City University Hospital Medical Center
Masaaki Kondo	Yokohama City University Hospital Medical Center
Shinichi Ohkawa	Kanagawa Cancer Center
Takeshi Suda	Niigata University Medical and Dental Hospital
Toru Ishikawa	Saiseikai Niigata Daini Hospital
Tatsuya Yamashita	Kanazawa University Hospital
Yoshitaka Inaba	Aichi Cancer Center Hospital
Yozo Sato	Aichi Cancer Center Hospital
Yukio Osaki	Osaka Red Cross Hospital
Takumi Igura	Ikeda Municipal Hospital
Toshihito Seki	Kansai Medical University Takii Hospital
Masatoshi Kudo	Kinki University Hospital
Kiyohide Kioka	Osaka City General Hospital
Eiji Mita	National Hospital Organization Osaka National Hospital
Shuhei Nishiguchi	Hyogo College of Medicine
Yoshiki Suginoshita	Kobe City Medical Center General Hospital

Mikiya Kitamoto	Hiroshima Prefectural Hospital
Hiroshi Aikata	Hiroshima University Hospital
Takahiro Yamasaki	Yamaguchi University Hospital
Takuji Torimura	Kurume University Hospital
Hiroaki Nagamatsu	Yame General Hospital
Seigou Abiru	National Hospital Organization Nagasaki Medical Center
